# A Multipurpose CMOS Platform for Nanosensing

**DOI:** 10.3390/s16122034

**Published:** 2016-11-30

**Authors:** Alberto Bonanno, Alessandro Sanginario, Simone L. Marasso, Beatrice Miccoli, Katarzyna Bejtka, Simone Benetto, Danilo Demarchi

**Affiliations:** 1 Center for Sustainables Futures@PoliTo, Istituto Italiano di Tecnologia, C.so Trento 21, 10129 Torino, Italy; ing.albertobonanno@gmail.com (A.B.); Katarzyna.Bejtka@iit.it (K.B.); 2 Electronics Design Laboratory (EDL), Istituto Italiano di Tecnologia, Via Melen 83b, 16152 Genova, Italy; 3 CNR-IMEM, Parco Area delle Scienze 37, 43124 Parma, Italy; simone.marasso@polito.it; 4 Department of Electronics and Telecommunication, Politecnico di Torino, C.so Duca degli Abruzzi 24, 10129 Torino, Italy; beatrice.miccoli@polito.it (B.M.); danilo.demarchi@polito.it (D.D.); 5 χ-Lab Materials and Microsystems Laboratory, DISAT, Politecnico di Torino, Via Lungo Piazza d’Armi 6, 10034 Chivasso, Italy; simone.benetto@polito.it

**Keywords:** CMOS interface, multipurpose sensing platform, CMOS post-processing, nanowires, nanosenors

## Abstract

This paper presents a customizable sensing system based on functionalized nanowires (NWs) assembled onto complementary metal oxide semiconductor (CMOS) technology. The Micro-for-Nano (M4N) chip integrates on top of the electronics an array of aluminum microelectrodes covered with gold by means of a customized electroless plating process. The NW assembly process is driven by an array of on-chip dielectrophoresis (DEP) generators, enabling a custom layout of different nanosensors on the same microelectrode array. The electrical properties of each assembled NW are singularly sensed through an in situ CMOS read-out circuit (ROC) that guarantees a low noise and reliable measurement. The M4N chip is directly connected to an external microcontroller for configuration and data processing. The processed data are then redirected to a workstation for real-time data visualization and storage during sensing experiments. As proof of concept, ZnO nanowires have been integrated onto the M4N chip to validate the approach that enables different kind of sensing experiments. The device has been then irradiated by an external UV source with adjustable power to measure the ZnO sensitivity to UV-light exposure. A maximum variation of about 80% of the ZnO-NW resistance has been detected by the M4N system when the assembled 5 μm × 500 nm single ZnO-NW is exposed to an estimated incident radiant UV-light flux in the range of 1 nW–229 nW. The performed experiments prove the efficiency of the platform conceived for exploiting any kind of material that can change its capacitance and/or resistance due to an external stimulus.

## 1. Introduction

Nanotechnology is the new frontier of research in several fields including sensing applications. In the recent years, material scientists are conceiving atomic structures and functionalized materials able to interact with the environment at nanoscale. Such nanostructures, having a remarkable sensitivity are good candidates for the new generation of extremely sensitive sensing devices, and are often called “nano-sensors”. Semiconductor nanowires, such as Si-NWs [1], ZnO-NWs [2] or InAs-NWs [3], are considered promising materials for sensing. The large variation of their electrical properties, related to the small sensing quantity detected at nanoscale, corresponds to high sensitivity. In order to completely exploit the sensitivity of these passive materials, it is essential to design a custom Read-Out Circuit (ROC) that must translate the electrical characteristics of these materials to digital signals compliant with standard electronic devices [4]. The size of the nanomaterial is nowadays comparable with standard CMOS electronics [5]. Indeed, during the years following Moore’s Law, the silicon fabs have continued to reduce the size of CMOS transistors, reaching dimensions comparable with most of the nanomaterials developed in the last few decades. For this reason the assembling of the sensing material onto the read-out electronics makes them even more interesting, enabling the fabrication of a high-accuracy and low-noise integrated sensing devices [6,7]. At research stage, semiconductor NWs (e.g., Si-NWs) have been already integrated with CMOS technology during the standard fabrication process and experimented as new Field Effect Transistors [8]. Considering the sensing purposes, the nanomaterial should be exposed to the external agent and therefore it has to be assembled onto the top electrodes of the CMOS technology using post-processing techniques [9]. For instance, some techniques involve the direct growth of nanowires on metal microelectrodes using seeds or self-assembly layers [10]. Unfortunately, these methods are not always compliant with post-processing on CMOS because of the need of chemical processes at high temperature that can cause rediffusion of the active area of CMOS transistors or deformations in the metal of interconnections. An easier technique is based on the drop casting of NWs onto a large area of interdigitated microelectrodes [11]. The probability of a successful integration is only proportional to the metal electrodes area and the number of nanowires per area. Alternatively, the electrodes can be post-fabricated after the nanowires dispersion onto a silicon substrate. In this case a low concentration of nanomaterial is randomly deposited onto an insulating substrate (usually by solvent droplet evaporation) and then, after a SEM imaging, the best nanowire in terms of dimension, length and position is electrically contacted by Focused Ion Beam (FIB) deposition or by 3D inkjet printing of conducting material [12]. However, this integration method is not suitable for a reliable, optimized and low-cost fabrication of a nanosensing device, because it would require customized additional lithography masks or advanced 3D printing technique for depositing post-fabricated metal electrodes in order to connect the NWs with the CMOS electronics [13,14]. A renowned and extremely low-cost technique to drive NWs deposition is the dielectrophoresis (DEP), which involves the use of a non-uniform electric field to orient and attract the nanowires, suspended in liquid solution, towards the region with highest electric field (i.e., the metal electrodes where the DEP signal is applied) [15,16,17]. The Micro-for-Nano (M4N) approach here described exploits such DEP technique to assembly single NWs across the top microelectrodes of a CMOS silicon chip, following a Multi-Electrode Array (MEA) topology. It also includes an additional CMOS post-processing step to improve the electric contact between the semiconductor nanowires and CMOS microelectrodes. Indeed, CMOS technology usually allows aluminum as top electrodes (AlTEs) which can easily oxidize if exposed to air before nanowires assembly. As the direct assembly of semiconductor NWs by DEP implies a small contact area between the deposited material and the AlTEs, the growth of an insulating layer as Al_2_O_3_ has to be avoided. For that reason, our fabrication process of M4N prototypes includes a low-cost gold-metalization of the AlTEs. The designed System-on-Chip (SoC) [18], connected to a microcontroller-based platform for chip configuration and real-time data processing, is composed by 8 DEP generators and 24 ROCs and supports both the assembly phase and the sensor measurements within all the multielectrode array, implementing an innovative portable device for nanosensing. In the next sections we will describe in details some advantages of the M4N approach, the low-cost fabrication steps to obtain a M4N prototype-chip with gold microelectrodes, and the complete system for real-time monitoring of a nanosensor array, including some results on ZnO-NWs assembled on M4N chip and used as UV sensors.

## 2. The Micro-for-Nano Approach

**Figure 1:**
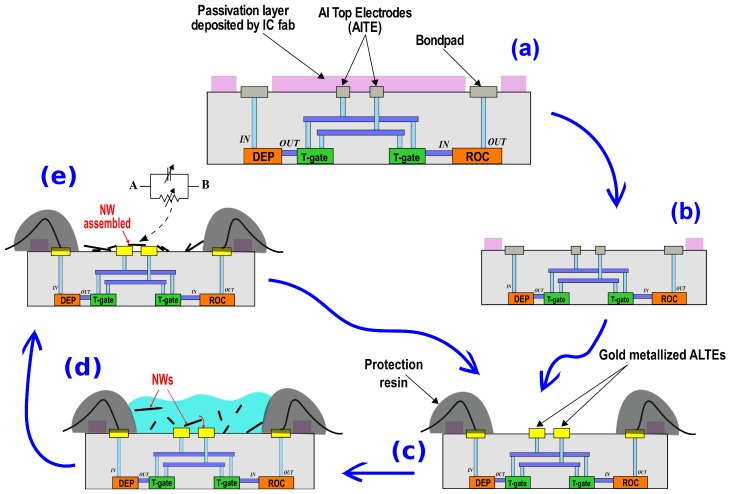
The complete process flow to enable the integration of semiconductor NWs onto M4N chip: (**a**) passivation removal; (**b**) gold plating of AlTEs, pads bonding and resin deposition for bondwire protection; (**c**) deposition of NWs by DEP; (**d**) assembled NWs can be used as nanosensors; (**e**) optional removal of assembled NWs by ultrasonic bath to reuse the M4N chip.

The conceived M4N system has been designed to deliver an easy-to-use and reliable platform for nanosensing. The M4N chip, custom designed with 130 nm CMOS technology, is the core of the whole system [18]. It includes the CMOS electronics designed for driving the NWs assembly and for reading-out their electrical properties. The complete process flow, needed to integrate nanosensors onto a M4N chip, is depicted in Figure 1. Usually, a chip designed in CMOS technology is protected by a passivation layer deposited by silicon fabs and composed of Silicon Nitride (Si_3_N_4_) and Phosphosilicate Glass (PSG). In our M4N chip, the AlTEs, designed for the nanomaterial assembly, are thus covered by this passivation film, as shown in Figure 1a. The dry etching of the passivation layer, implemented using the Reactive Ion Etching (RIE) machine with SF_6_ gas, is the first step needed to expose the AlTEs and to allow the deposition of nanomaterials (see Figure 1b).

Consequently to air exposure, the aluminum surface easily oxidizes, i.e., forming an insulating layer which worsen the electrical contact with the NWs when they are deposited. Thus, a chemical process is required to remove the oxidation layer and to cover the CMOS electrodes with gold, hence enabling the low current flow through the small contact area between gold electrodes and NWs. After this chemical treatment and metalization, the M4N chip can be bonded on a custom printed circuit board (PCB), covering the pad ring with a insulating resin in order to protect bondwires, as shown in Figure 1c.

Hereafter, the M4N chip can be programmed for enabling the NWs assembly using the DEP technique, as depicted in Figure 1d. Eight digitally-controlled triangle-wave oscillators [18] on-chip, singularly connected to a small cluster of AlTEs, are used to generate the DEP signal with adjustable oscillation frequency in the 50 kHz–1 MHz range. This AC signal generates an electric field between each couple of AlTEs, inducing an attractive force between the NWs suspended in solution and the specific microelectrodes. Exploiting the DEP technique and the integrated array of DEP generator, a customized layout of nanosensors on-chip (i.e., the exact position of NWs on M4N chip) is allowed.

Once the DEP process is completed and the required NWs are aligned between the AlTEs couples, the integrated ROC can measure the nanomaterial impedance [18] and monitor it during the sensing application (see Figure 1e). The NWs, which have not been connected to AlTEs during DEP process, stick to the SiO_2_ of the chip surface with no consequence for sensing. Assembled NWs are firmly attached to the electrodes. A chemical attack or an ultrasonic bath is necessary to remove them from M4N chip. Such strong but reversible chemical bond allows subsequent NWs depositions without affecting the previous ones. The following section reports a detailed description of the fabrication steps of M4N chip. This includes the removal of the passivation layer and gold-metalization of the electrodes required for the integration of the NWs onto the AlTEs.

## 3. Results and Discussions

### 3.1. The Low-Cost CMOS Post-Processing

**Figure 2:**
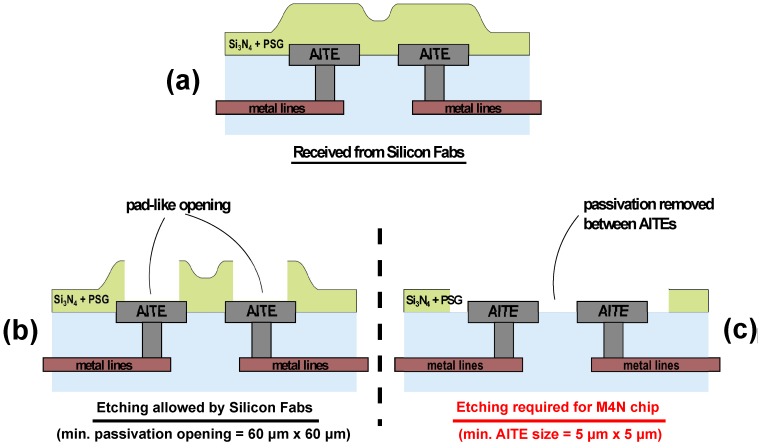
Etching of passivation film onto the M4N chip: (**a**) the chip received from semiconductor fab; (**b**) the standard solution for opening windows and (**c**) the required M4N solution for nanowire assembly.

The M4N chips have been fabricated using standard 130 nm CMOS technology and exploiting Multi-Project Wafer strategy to limit fabrication costs. As mentioned in the previous section, after the deposition of the top metal layer, the processed silicon wafer is covered by the passivation layer, usually composed by two layers of Si_3_N_4_ and PSG, which protects the surface of the chip from dust and impurities (as depicted in Figure 2a). These layers are undesired because the nanomaterial must be in contact with the metal electrodes. The standard CMOS process flow allows the opening on the surface of specific windows in order to access the aluminum layer underneath the passivation. This is the same approach commonly used for opening I/O pads. However, for standard design rules in 130 nm process, the openings have to be larger than about 60 μm × 60 μm and smaller than the underlying metal area, leaving the passivation between the AlTEs (see Figure 2b). These conditions limit the maximum number of nanosensors on-chip. Moreover, the integration of NWs onto the M4N chip requires the removal of the passivation layer even between each couple of electrodes, as shown in Figure 2c. For final preparation of the chips and to avoid very expensive specific steps including design of the additional lithographic masks during standard CMOS process, the naked M4N chips have been post-processed using a RIE machine, wherein the chip surface is subjected to a chemical and physical attack. A RIE STS 320 was employed to perform the Si_3_N_4_ and PSG etching, whereas the aluminum acts as stop etch layer. The details of the etching condition are the following: 16 sccm of SF_6_ gas flow, 75 mTorr of chamber pressure, 150W of RF power. During the RIE process, the pad-ring has been protected using a stainless steel hard mask to preserve Si_3_N_4_ between I/O pads as shown in Figure S1.

The whole etching process takes about 13 min to completely remove passivation from the chip surface and can be performed on several chips at the same time.

**Figure 3:**
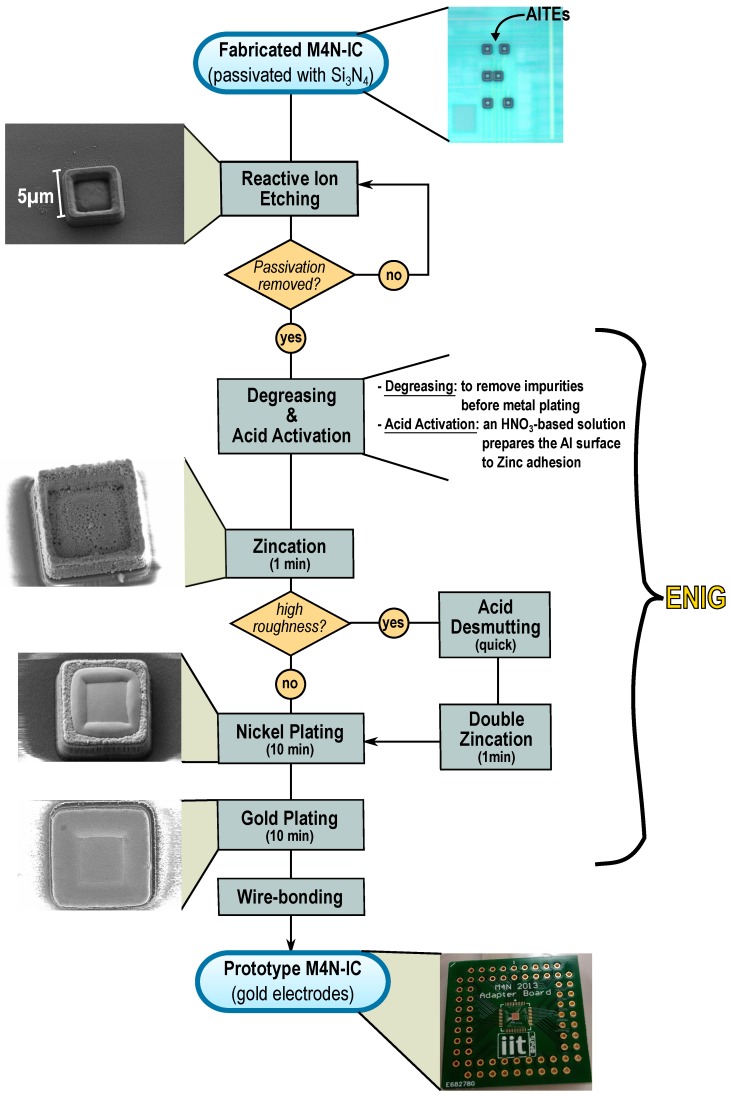
Electroless post-processing on M4N chip needed to cover the AlTEs with gold, enhancing the electrical contact between nanomaterials and microelectrodes.

After RIE steps, the surface of the AlTEs has been analyzed by Field Emission Scanning Electron Microscopy (FESEM) and an accurate Energy Dispersive Spectroscopy (EDS) has been performed to assure that the protection layers have been removed. FESEM charcterization showed that the morphology of the pads was preserved after the RIE process, as also shown in the first step of the process-flow reported in Figure 3. The complete removal of the passivation layer was confirmed by EDS that detected the presence of Al and oxygen only (Supplementary Materials Figure S2). As mentioned above, a metalization of AlTEs with gold is required to avoid electrodes oxidation before NWs assembly. One of the most used methods is the electrodeposition [19], where the electrodes are placed in an electrolytic bath and a differential voltage is imposed between the donor electrode (i.e., cathode) and the AlTEs (i.e., anode). Such method implies a direct access to all the AlTEs to ground them and to allow a current flow, which cannot pass through unpolarized small CMOS transistors. In our M4N fabrication flow, we implemented an electroless plating based on chemical reactions using low-cost commercial solution (Technic Inc.) conceived for different market (e.g., jewelry plating). The used technique is called Electroless Nickel Immersion Gold (ENIG) [20,21] and it is usually implemented in CMOS technology to obtain gold-metalized pads for flip-chip bonding [22,23].

Considering the micrometer scale of the gap between AlTEs (i.e., 2 μm), the timing and the concentration of the chemical compounds for the ENIG process have to be accurately optimized in order to obtain slow and stable growth rates. The first step of the procedure implies the surface treatment using an alkaline solution Technic TSC 1500 at 68 °C for chemical degreasing of the aluminum, followed by an acid attack at room temperature, containing Technic Remova 1700 and 1% of HNO_3_. The acid attack is used to remove both the residuals of silicates from previous chemical reaction and the Al_2_O_3_ grown on top of AlTEs. Each step of our ENIG process, reported in Figure 3, is followed by a washing of the M4N chip in bidistilled water for 1 min. The electroless gold deposition onto aluminum electrodes needs adhesion layers to be effective. The standard plating procedure implies a zincation treatment of the aluminum surface, i.e., a galvanic displacement reaction between Zn ions and Al atoms, to avoid the Al_2_O_3_ creation by depositing a thin Zn layer on top. For that purpose, we used the commercial solution Technic EN Zincate at room temperature. Moreover, the thin zinc layer deposited onto AlTEs acts as a catalytic layer for the Au electroless deposition. A single zincation takes about 1 min but the electrode surface could still appear rough and thus not optimized for the following Ni/Au deposition. A second zincation, preceded by a quick immersion into a 1% HNO_3_ solution for desmutting, is suggested to reduce the roughness of the Zn layer [24]. Since the zincation process is a displacement reaction between Zn and Al atoms, the AlTEs are now covered by a thin layer of Zn atoms and both elements are detected during the EDS analysis, as shown in Supplementary Materials Figure S3. Nickel is then deposited using a solution based on Technic ENickel AT 5000 heated to 85 °C where the M4N chip is immersed for 10 minutes. As shown in Figure 3, in this case the process is additive and the nickel is successfully deposited on the surface. A 10 min immersion in a solution containing Technic OROMERSE SO heated to 73 °C is needed to cover the microelectrodes with gold. The results for each step are shown in Figure 3 and in Supplementary Materials Figure S3. The whole process to cover the AlTEs with noble metal costs less than 1 € per chip, thus being highly sustainable for low-cost experiments.

**Figure 4:**
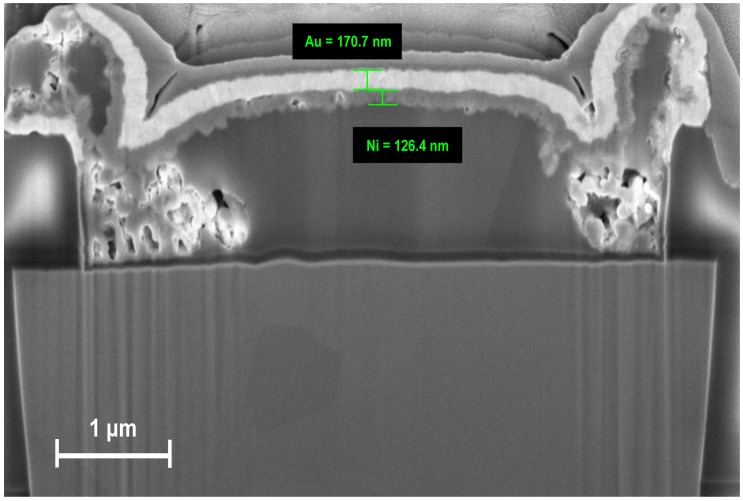
Cross-section FESEM image of the M4N chip. AlTEs are covered by thin layer of Nickel (126 nm) and Gold (170 nm). The estimated growth-rate is about 13 nm/min and 17 nm/min, respectively.

The growth rate and the quality of the deposited metals has been verified by analyzing the cross-section of a prototype M4N chip, created with the use of the Focused Ion Beam (FIB). The cross-section view, shown in Figure 4, confirms the presence of the metalization layers. The Zinc layer is not visible, because it confirms only a thin coverage of the aluminum electrodes. The growth rate for Nickel and Gold are estimated to be 13 nm/min and 17 nm/min respectively. The top visible layer is platinum, deposited in-situ on a single electrode for the preparation of the FIB cross-section.

Once the metalization is completed, the M4N chip can be bonded on a PCB-adapter and connected to the microcontroller-based system for being programmed and then used for the sensing experiments.

### 3.2. The Configurable Micro-for-Nano Sensing-Array

**Figure 5:**
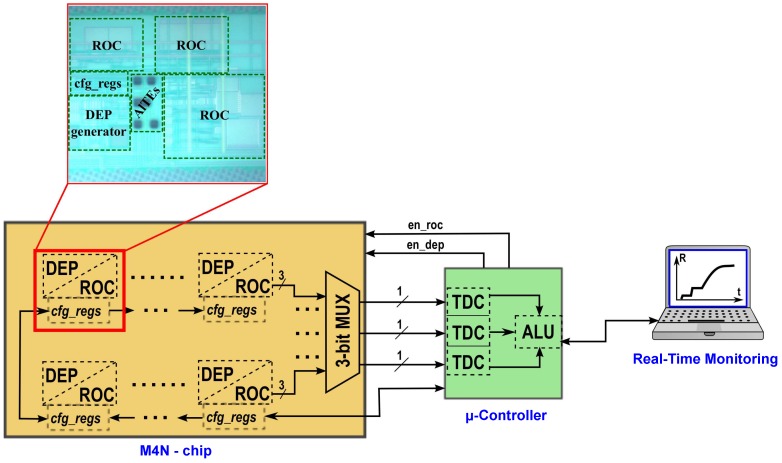
The block diagram of the whole system for nanosensing. The microcontroller-based platform is used to configure the M4N array, to convert the quasi-digital outputs to N-bit values using Time-to-Digital Converters (TDCs) and to redirect the elaborated data to the workstation in real-time. ALU: Arithmetic Logic Unit, cfg_regs: configuration registers of the M4N array.

Each of the 8 elements of the M4N array, described in Figure 5, is a small System-on-Chip (SoC). quasi-digital ROCs, parasitics at the input. The SoC is composed by 3 couples of facing gold AlTEs, a DEP generator and 3 quasi-digital ROCs. During the NWs assembly phase, the AlTEs are all connected to the DEP generator, whereas each couple of AlTEs is only connected to a single ROC to reduce parasitics during sensing experiments. Each SoC is an independent element of the M4N array and it can be configured with different parameters (e.g., settings for DEP frequency, calibration values for each ROC), loading configuration data into 3 registers of 8-bit using a Serial Peripheral Interface (SPI)-like interface. Moreover, all register-banks in the array are connected in a JTAG configuration, so that all the registers in the M4N chip can be sequentially loaded using a single SPI interface.

Considering that each M4N array element can be differently configured, it is possible to conceive a specific nanosensors layout on top of the M4N chip. The NWs, suspended in solution, are indeed attracted towards the AlTEs only if the connected DEP generator is enabled before that the solvent completely evaporates. The unconnected NWs stick on the silicon oxide surface without any impact. Following this procedure, it is possible to iteratively assembly up to 8 different types of NWs (i.e., the number of independent element of the M4N array within the current version) creating an integrated multipupose sensor platform. Once the DEP process is completed, the AlTEs can be connected to the ROCs to measure the impedance of assembled NWs, monitoring any variation of the electrical parameters related to sensing properties at nanoscale. Despite the large nanosensor array, thus the large number of impedance converters, only a limited number of I/O pads can be reserved for the output of ROCs. In this version of the M4N chip, all the 24 outputs of the ROCs are managed by a multiplexer that routes only 3 signals at the same time to the external microcontroller. A time-multiplexing approach can be easily implemented, since the microcontroller can iteratively select, defining a custom time-window, which quasi-digital output has to be directly connected to the Time-to-Digital Converters (TDCs) for data elaboration.

The wide configurability of the M4N chip enables high flexibility of the sensing system, allowing a custom layout of nanosensors on-chip and a selection of the most performing sensors for data acquisition and elaboration.

### 3.3. Real-Time Portable Electronic Interface

The interface between the M4N chip and the commercial electronics is implemented using Arduino Due, a low-cost platform based on the ARM Cortex Sam3x microcontroller. This choice allows to conceive a plug-and-play solution to easily connect the M4N chip to a working station for data storage and real-time data plotting during experiments.

**Figure 6:**
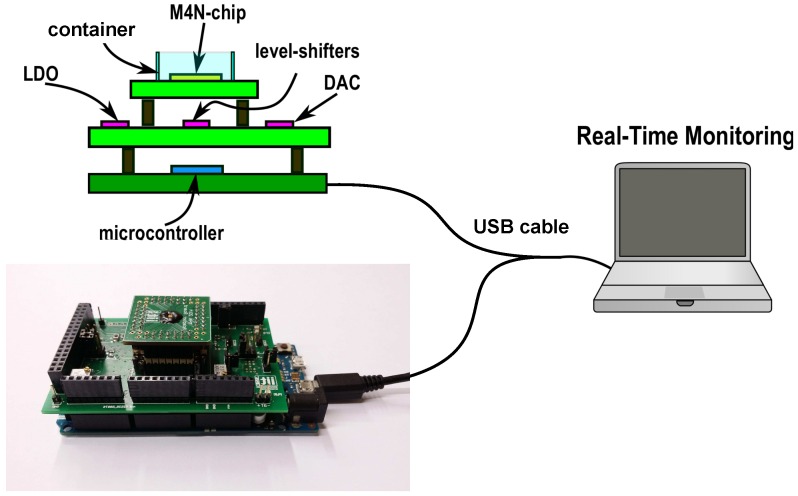
Entire sensing system consisting in three modules: the M4N chip (**Top**), an interface board (**Middle**) and the microcontroller platform (**Bottom**), which is directly connected to a notebook for data storage. Under the sketch, a photograph of the fabricated M4N system.

As previously mentioned, the quasi-digital ROC output is a square-wave signal with variable timing properties depending on the electrical characteristics of sensors [25]. As already described in [18], the Quasi-Digital Impedance Converter (QDIC) integrated into the M4N chip encodes the NW capacitance ($C_{NW}$) to the time at logic ‘0’ ($T_{0,NW}$) of the output signal, whereas the time at logic ‘1’ ($T_{1,NW}$) is directly proportional to the NW resistance ($R_{NW}$). This ‘quasi-digital’ approach allows to use low-complexity, low-power and low-voltage circuits for converting a large resistive and capacitive input range to 1-bit output signal. This solution is suitable for large arrays of sensing elements, where a high number of sensor data have to be routed to external units for elaboration. According to this approach, the information is encoded by events (i.e., the rising and falling edges of the square-wave defines $T_{0,NW}$ and $T_{1,NW}$) and the ROC outputs can be processed concurrently by several TDCs in parallel to extract the original information on $C_{NW}$ and $R_{NW}$. In the proposed M4N system, the TDCs are implemented using configurable blocks already available on microcontroller and active on external interrupts. Indeed, up to 8 independent timers can be programmed for measuring a delay between different interrupts (i.e., falling or rising edges of an input signal) with a maximum time resolution equal to the system clock period (i.e., 12.5 ns for the ARM Cortex Sam3x). Based on TDC the output value, the microcontroller can calculate the average value and standard deviation of $T_{0,NW}$ and $T_{1,NW}$, estimating the correspondant value of $C_{NW}$ and $R_{NW}$ by using equations described in [18]. As represented in Figure 5, 3 TDCs are configured to process the quasi-digital ROC outputs of the M4N chip in parallel. The whole system is depicted in Figure 6 and it includes the M4N chip, an interface module and the microcontroller platform connected via USB to a workstation for data storage. As mentioned in Section 3.2, the M4N chip is assembled as chip-on-board, using a footprint compliant to the standard PLCC68 socket.

**Figure 7:**
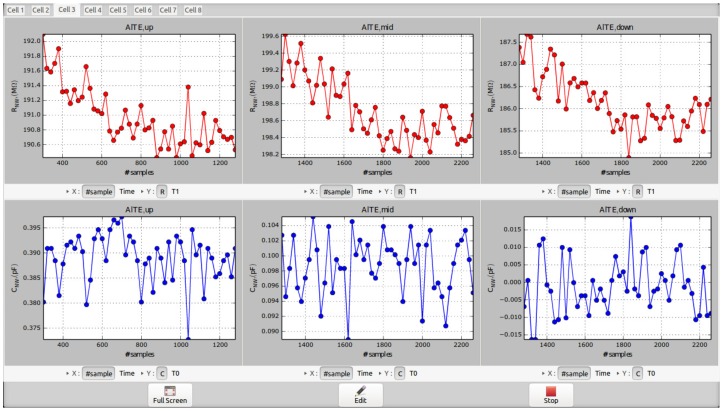
A screenshot of the developed graphical user interface needed for the real-time plot of the electrical properties (i.e., $R_{NW}$, $C_{NW}$) of the assembled semiconductor nanowires.

The bondwires are covered by black resin to protect them and an additional plastic ring can be mounted on M4N chip in case of bio-sensing measurements in liquid solution. This approach allows to consider the M4N chip as a “cartridge” for dedicated experiments. A prototype of the whole system and a detail on a single M4N chip assembled for bio-sensing are shown in Figure 6 and Figure S6, respectively. A user-friendly Graphical User Interface (GUI) has been developed to easily configure the M4N chip for the selected experiment and to plot in real-time the measurement data about the 3 nanosensors of a single M4N array element. The main commands for the M4N-SoC are used to define the custom layout of nanosensors on chip (i.e., where the DEP signal has to be enabled within the M4N array), to load the calibration settings for each ROC and to select which ROC output signal is redirected to microcontroller and plot in real-time. A screenshot of the window for real-time plot is shown in Figure 7, whereas the windows for settings and calibration are reported in Supplementary Materials Figure S5.

### 3.4. Experiments Using Single ZnO-NWs

Among semiconductor nanomaterials, ZnO nanowires are widely used since they are sensitive to mechanical stimuli [26], UV-light irradiation [2,27,28], chemical particles [29,30,31,32] and biological molecules [33].

**Figure 8:**
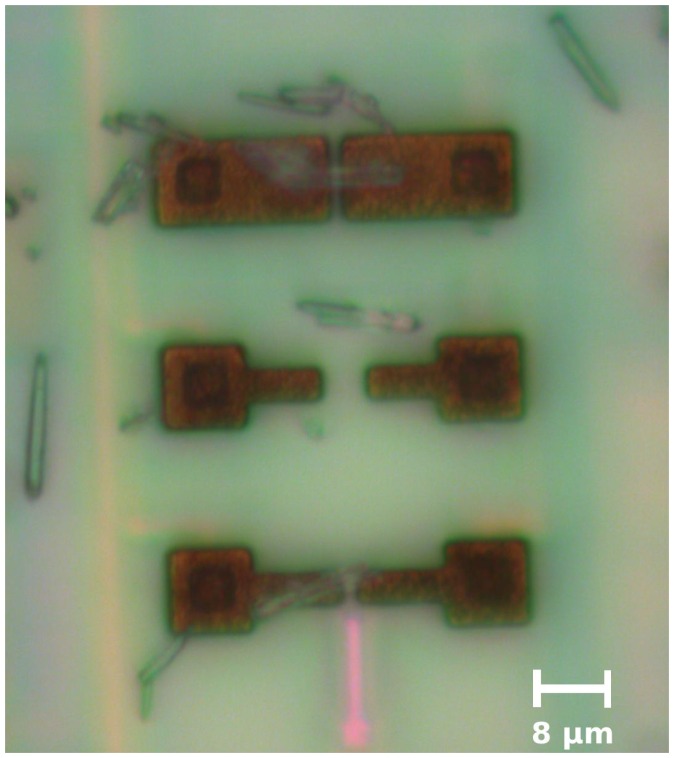
Example of a ZnO-NW successfully integrated onto M4N chip (upper electrode pair).

**Figure 9:**
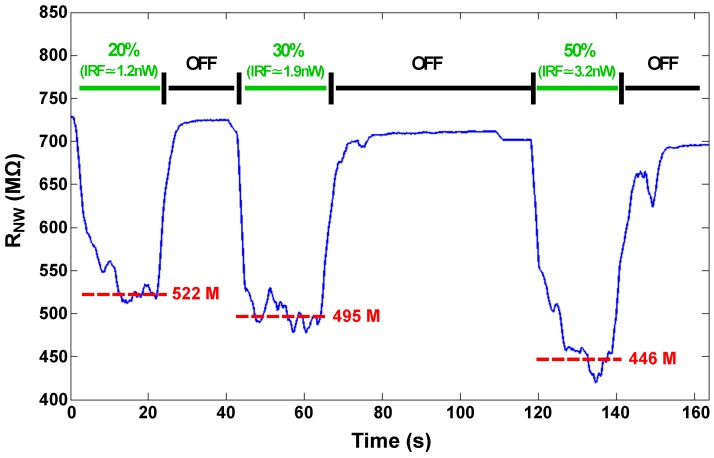
Real-time behaviour of the $R_{NW}$ of ZnO-NW${}_{A}$ when it is exposed to the UV source placed at 6 cm and working at 20%, 30% and 50% of the maximum irradiance power. The IRF-UV corresponds to about 1.2 nW, 1.9 nW, 3.2 nW, respectively.

Hence, we used ZnO nanowires to validate our approach. They have been assembled by DEP onto the M4N chip enabling only four DEP generators to drive the assembly of ZnO-NWs on a defined area of the chip. Successful electrical connections have been created between the ZnO-NWs and several couples of AlTEs of the M4N array like the one showed in Figure 8 in which one ZnO-NW has been successfully integrated between upper pair of electrodes. We chose two to make further discussion and we identify them as ZnO-NW${}_{A}$ and ZnO-NW${}_{B}$. Under dark conditions, the resistance of ZnO-NW${}_{B}$ is about 330 MΩ, whereas the $R_{NW}$ measured for ZnO-NW${}_{A}$ is about 700 MΩ. A LightningCure LC8 UV lamp, with adjustable power irradiance and emission wavelenght of about 365 nm, has been used to evaluate the sensitivity of a single ZnO-NW and to validate the M4N approach. As described in the UV lamp datasheet, by considering a sample placed at distance d = 1 cm, the emitted radiant flux is focused by a specific light guide on a restricted area of about 1 cm${}^{2}$ with a measured maximum irradiance of about 4500 mW/cm${}^{2}$. The enlighted area slightly increases if the sample is placed at higher distance, whereas the irradiance decreases as 1/d${}^{2}$. Assuming that the irradiance is constant on the whole enlighted area and considering a single ZnO-NW with size of about 5 μm × 500 nm assembled on the M4N chip, the Incident Radiant Flux (IRF-UV) on the ZnO-NW is only a small part of the emitted UV light flux and proportional to its surface (i.e., 7.85 μm${}^{2}$). In particular, since the power of the UV lamp can be adjusted from 1% to 100% of the nominal power, the estimated IRF-UV varies from 2.29 nW to 229 nW for a ZnO-NW placed at 1 cm The results of the On/Off experiment are shown in Figure 9, where the UV light source is alternatively switched off and on at 20%, 30% and 50% of the maximum irradiance power. The reported data have been already weighted by considering the influence of the UV irradiation on the CMOS circuit, estimated during tests reported in the Supplementary Materials. Once the UV lamp is switched on, the $R_{NW}$ decreases due to the contribution of photogenerated current.

The higher the UV light incident the more electron-hole pairs will be generated in the material, i.e., the higher will be the total current. Specifically, the oxygen ions (O^2−^) naturally adsorbed on the ZnO NW surface will attract the photogenerated holes, while the electrons will contribute to the current in the conduction band [34,35]. Although the assembled NWs have different electrical characteristics, they are both sensitive to the UV light with similar trend. The sensitivity has been calculated a piecewise linear approximation of the experimental curves within two regions delimited by an ideal knee point at 20 nW. Both NWs show best sensitivity for an estimated IRF-UV lower than 20 nW.

**Figure 10:**
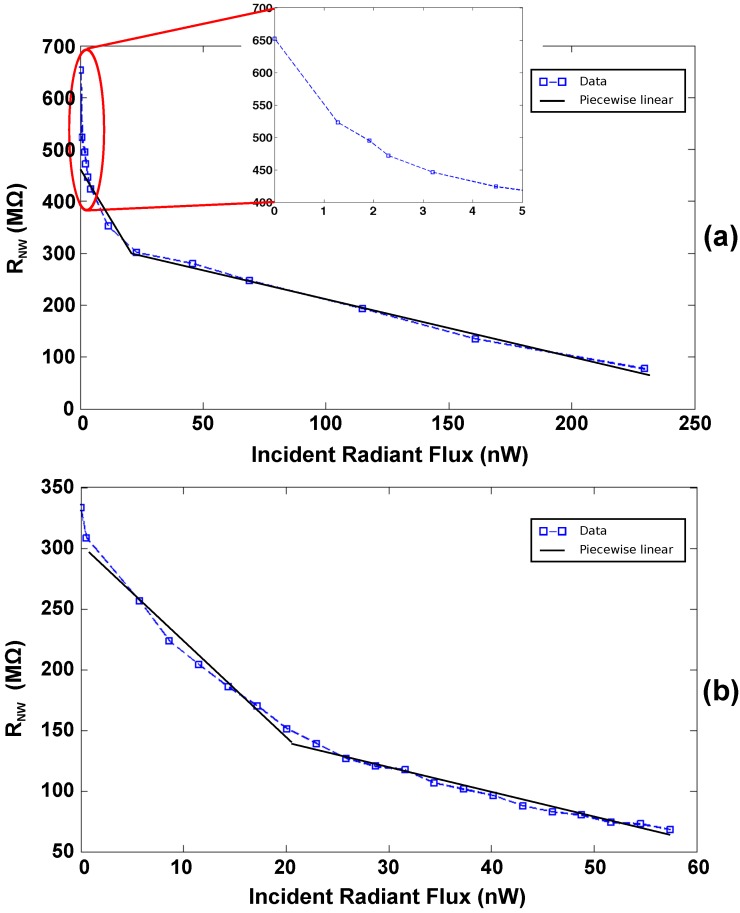
Variation of the $R_{NW}$ depending on the Incident UV-light Radiant Flux (IRF-UV) for ZnO-NW${}_{A}$ (**a**) and ZnO-NW${}_{B}$ (**b**) in the same M4N array. During experiments reported in (**a**), the M4N chip has been placed at 1 cm and 6 cm from the UV source, whereas in (**b**) the M4N chip has been fixed at 2 cm.

Several experimental data for the ZnO-NW${}_{A}$ are reported in Figure 10a and the linear approximation of the nanosensor sensitivity to UV-light is about −10.21 MΩ/nW for IRF-UV < 20 nW and about −1.08 MΩ/nW for IRF-UV > 20 nW. Similarly, the other ZnO-NW assembled on the same M4N chip, whose experimental data are plotted in Figure 10b, shows a sensitivity to UV-light of about −8.04 MΩ/nW for IRF-UV < 20 nW, whereas it decreases to about −2.23 MΩ/nW for higher UV intensity. The UV experiment is only reported as proof of concept for the M4N system validation, since the designed platform has to be considered as an essential support for implementing nanowire-based sensing in different field (e.g., chemical or biomedical). Indeed, the electronics integrated on the M4N chip has been already used for real-time biosensing, as described in detail in [36], where the measurement on capacitance reveals the adhesion of BSA protein on single functionalized ZnO-NW assembled on external gold nanogap device. Performing the same experiment on the M4N surface will increase the sensitivity due to an evident and expected noise reduction.

## 4. Conclusions

A complete system for nanomaterial-based sensing platform has been described in detail. The system is composed of both hardware and software building blocks. The hardware core is represented by the 130 nm CMOS technology silicon chip with the microcontroller-based low-cost platform to interface the M4N IC. While, an easy configurable GUI was developed for real time data visualization. The ZnO-NWs are then successfully integrated across the AlTEs using the on-chip DEP generators, creating the electrical connections with the gold microelectrodes. Two integrated ZnO-NWs are then used as UV-light sensors. The results of these experiments show large variation of the $R_{NW}$ up to 80% of the nominal value, if the single ZnO-NW is exposed to different incident UV light radiant flux in the nW range. The average sensitivity of the two NWs to UV light can be calculated in two IRF-UV range separately. Indeed, the UV sensitivity for ZnO-NW${}_{A}$ and ZnO-NW${}_{B}$ is about −10.21 MΩ/nW and −8.04 MΩ/nW for IRF-UV < 20 nW, whereas it is about −1.08 MΩ/nW and −2.23 MΩ/nW for IRF-UV > 20 nW, respectively. The slight difference of $R_{NW}$ and UV sensitivity between ZnO-NW${}_{A}$ and ZnO-NW${}_{B}$ probably depends on the size of NWs or on the electrical connection with AlTEs.

The proposed system is not only conceived of for experiments on light exposure, but also suits for experiments in different fields (e.g., chemical or biomedical), depending on the material and on the chemical functionalization of nanowires assembled onto the M4N chip. Thus the described M4N system represents a step forward for an ease-to-use nanowire-based sensing platform with integrated electronic circuits.

## Supplementary Materials

The Supplementary Materials are available online at http://www.mdpi.com/1424-8220/16/12/2034/s1.
