# Supplementary material for: A Multipurpose CMOS Platform for Nanosensing
Source: Sensors (Basel). 2016 Nov 30;16(12):2034. doi: 10.3390/s16122034 (PMC5191015; doi:10.3390/s16122034)
Supplement: Supplementary file 1 [file sensors-16-02034-s001.pdf]

Supplementary Materials: A Multipurpose CMOS Platform for Nanosensing

Alberto Bonanno, Alessandro Sanginario, Simone L. Marasso, Beatrice Miccoli, Katarzyna Bejtka, Simone Benetto and Danilo Demarchi

1. CMOS Post-Processing for Gold Plating of Microelectrodes

Bondpads were protected from RIE attack by means of a hard mask made in stainless steel visible in Figure S1. Realized mask has four slots for processing four chip in the same RIE session. When a naked chip is covered by the mask (the mask is placed in contact), the bondpads area are covered, thus only the central part of the chip, affected by the RIE attack, is removed from the passivation layer.

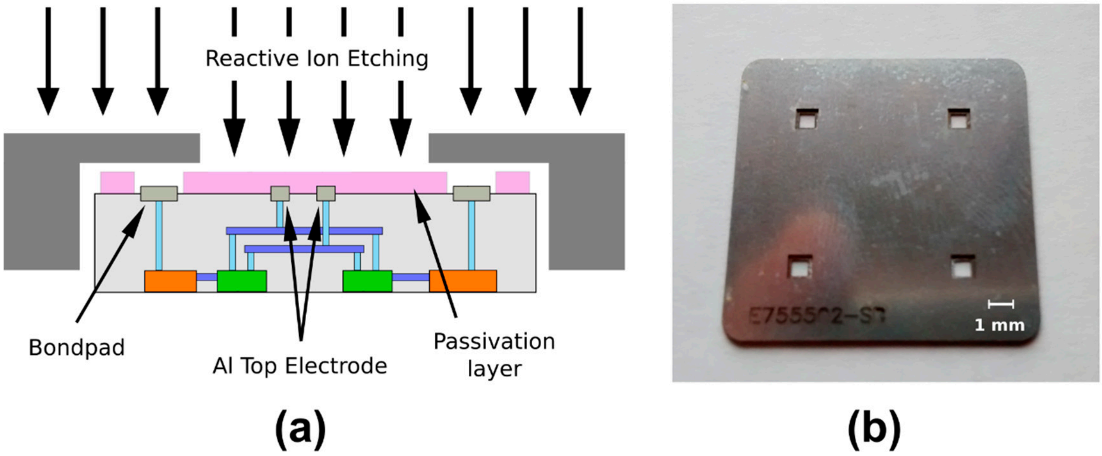

Figure S1. (**a**) Sketch of stainless steel mask used to cover the bondpads from RIE attack; (**b**) Image of the realized stainless steel mask.

Figure S2 shows the results of EDS analysis performed using FESEM. Such analysis was performed to understand when the passivation layer was completely removed by RIE and to observe if the morphology of the electrode was preserved.

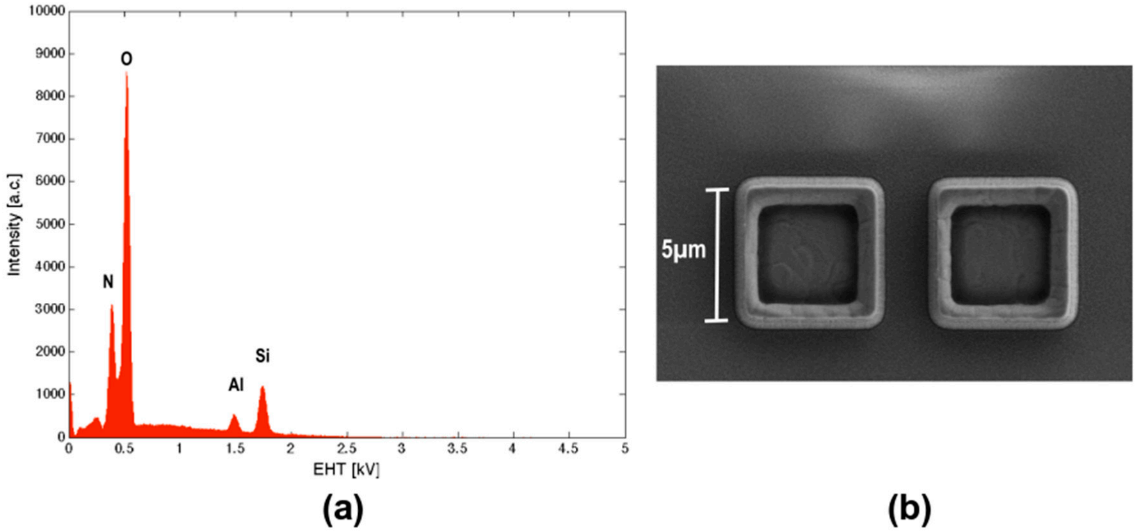

Figure S2. (**a**) Results of EDS analysis on M4N chip as it is received from silicon fab; (**b**) Fesem image of passivated electrodes on which EDS analysis was performed.

Once the Si3N4 and the PSG have been removed, the EDS analysis was also used to optimize the steps of the ENIG process on M4N chip. Figure S3a is the starting point of the process and the analysis confirms that the aluminum electrode is exposed and slightly oxidized. Figure S3b shows the presence of a thin layer of Zn after a double zincation, but the Al can be also detected below.

The Nickel and Gold deposition are additive processes, as also demonstrated by EDS analyses reported in Figure S3c,d where only the material on top can be detected. The thickness of the deposited layers can only be estimated after a cross-section of the M4N chip, as described in the main text.

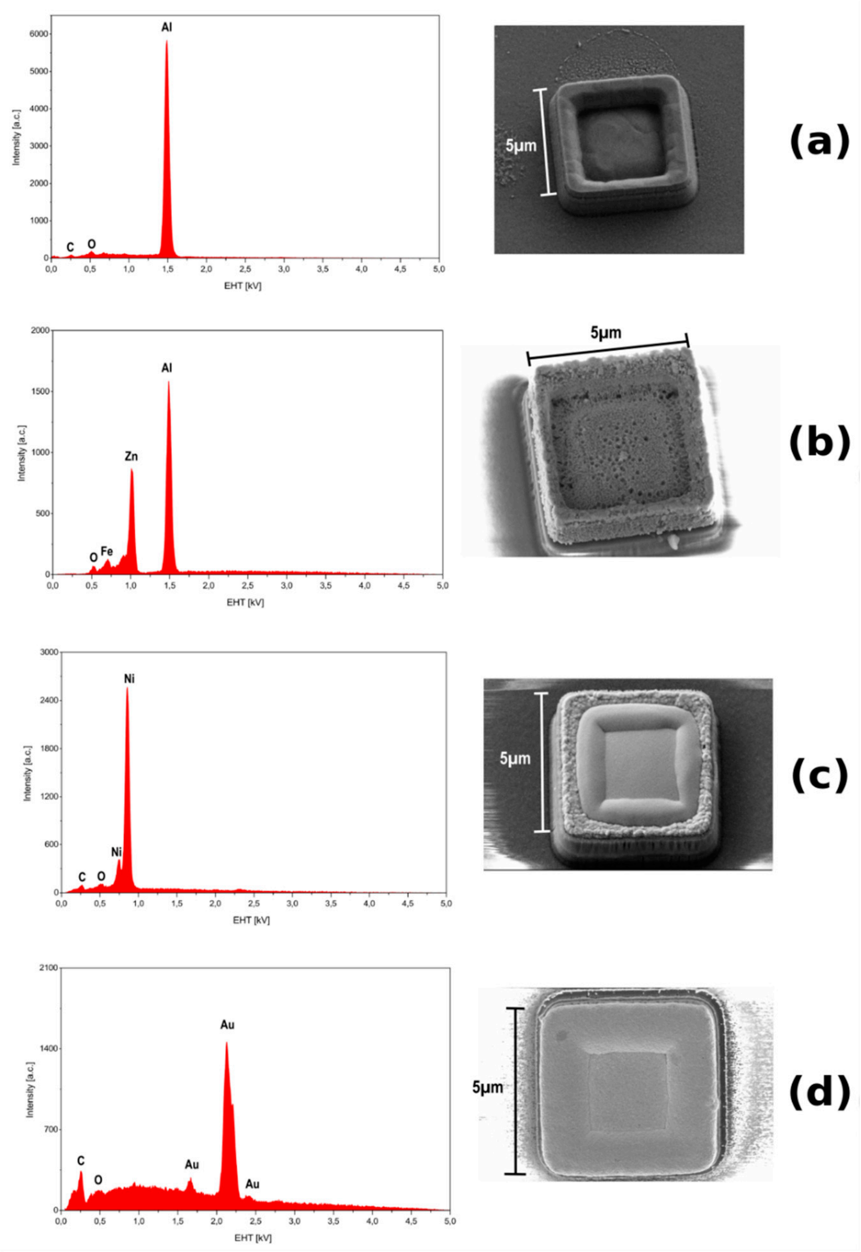

Figure S3. Results of EDS analysis after each step of the electroless process: RIE attack (**a**); Aluminum oxide removal and Zn deposition (**b**); Nickel (**c**); and Gold (**d**) plating.

2. Real-Time Experiments under UV-Light Exposure

As described in the reported experiments using the proposed M4N approach, the ZnO-NWs are assembled onto gold-metalized microelectrodes placed just on top of the CMOS read-out circuit. As reported in literature [1,2], P-N junction of doped silicon is also sensitive to light in a large wavelength range (e.g., CMOS photodiode). During real-time experiments at different UV

irradiance power, the whole chip is exposed to an intense UV light flux and a small variation of the ROC output has been also detected even where the NWs were not connected. Indeed, the ROC circuit, exposed to UV light flux even if attenuated by thick silicon oxide layer, can be affected by leakage current variation.

As explained in [3] and in this work, the leakage current estimation during characterization steps is fundamental for accurate RNW calculation. The designed ROC reads-out an equivalent resistance and capacitance at the input and encodes these values on time delays T1 and T0 of a square-wave generated at the ROC output. Small variations on T1 have been noticed, corresponding to a reduction of the maximum measurable resistance as proved by the behavior of the black curve in Figure S4.

Hence, considering a standard upper limit resistance (RMAX) of about 1 GΩ without any assembled NW on top of the ROC, the corresponding average leakage current (Ileak) is about 700 pA (i.e., Ileak = VROC, stim/RMAX). The decrease of RMAX observed during experiments suggests an increase of about 175 pA of Ileak in case of 1.2 nW of UV irradiation power. Under dark conditions, the equivalent INW is about 1 nA considering that the applied stimulation voltage Vstim, AC applied by ROC is 100 mV peak-to-peak with a DC value of about 700 mV. Under IRF-UV ≈ 1.2 nW, the RNW decreases from 700 MΩ to 460 MΩ (i.e., INW ≈ 1.521 nA) that corresponds to an increase of 521 pA. Actually, if considering leakage current as a constant value, there would be an overestimation of about 175 pA. Subtracting this value, we obtain to INW ≈ 1.346 nA and, thus, to RNW ≈ 520 MΩ.

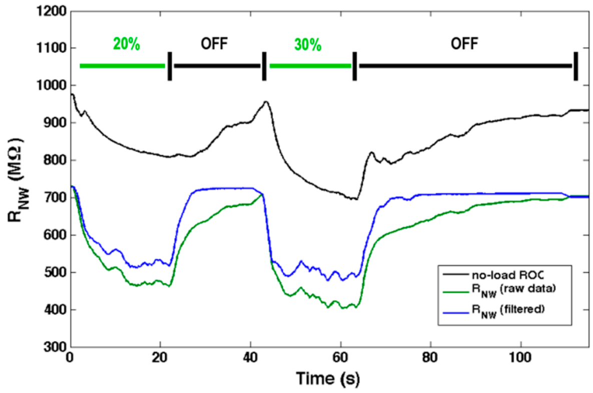

Figure S4. Estimated RNW under UV-light exposure: the maximum measurable resistance (**black curve**), the raw data (**green curve**) and the filtered data (**blue curve**) of RNW.

For this reason, a single ROC in the array has been considered as reference, disconnecting the couple of AlTEs from the underlying circuit and estimating the increase of Ileak due to UV light. In real-time data processing, such contribute has been subtracted from the measurement results obtained by the assembled ZnO-NWs used as UV sensors. The normalized blue curve is reported in Figure S4. This effect is almost negligible for more conductive ZnO-NWs (e.g., ZnO-NWB).

In [3], such compensation was not required because the NWs were placed on an external passive device (i.e., a gold electrode nano-gap) and not directly assembled on the silicon M4N chip. The excitation and relaxation time corresponding to the switching on and off of the UV source are now few seconds and the drift under constant irradiation is completely removed. This correction of the estimated RNW is needed only for experiments including UV light and large temperature variations, since these factor can induce small variation on leakage of ROCs. The characterization of the designed ROC under temperature variations is reported in [3]. Indeed, chemical or bio-sensing on M4N chip would not influence the ROC behavior.

3. Graphic User Interface for M4N System Control

Figure S5 shows two screenshots of GUI windows. Before launching the experiment, the user has to insert some calibration data (see Figure S5a) to set-up the tool and to optimize the RNW and

CNW calculation. Later, the assembly of NWs onto the M4N chip or an experiment using previously assembled NWs can be run, defining which circuits (i.e., DEP generators or ROCs) have to be enabled (see Figure S5b). The M4N system is now ready to use.

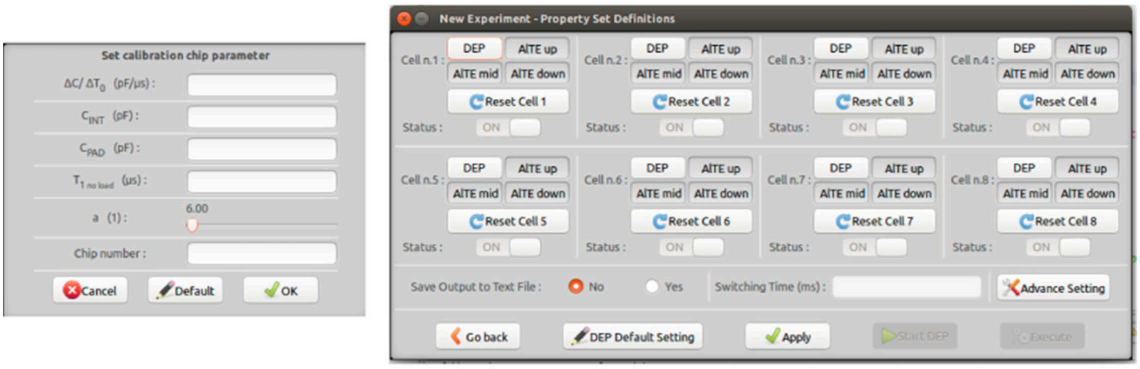

(a) **(b)**

Figure S5. Screenshot of the GUI windows for calibration data insertion (**a**); and for M4N array configuration (**b**).

4. M4N Cartridge for Biosensing Experiments

Figure S6 shows an example of how to use the M4N cartridge for biosensing experiment. Since most of biological experiments needs to be carried out into an acqueous environment, we sealed a plastic cuvette around the chip and we checked possible liquid leakage. Such cartridge is an example of the versatility of proposed platform.

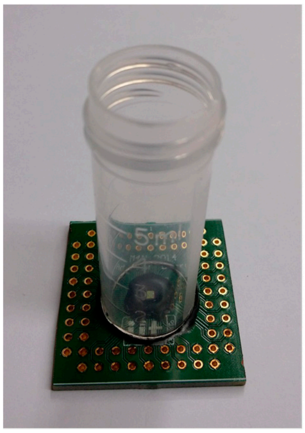

Figure S6. A M4N cartridge prepared for biosensing: the CMOS chip with the assembled NWs is sealed by a plastic container filled by the bio-solution to be analyzed.

References

1. Soo, L.J.; Hornsey, R.I.; Renshaw, D. Analysis of CMOS photodiodes. I. quantum efficiency. *IEEE Trans. Electron. Devices* **2003**, *50*, 1233–1238.
2. Manijeh, R.; Rogalski, A. Semiconductor ultraviolet detectors. *J. Appl. Phys.* **1996**, *79*, 7433–7473.
3. Bonanno, A.; Morello, M.; Crepaldi, M.; Sanginario, A.; Benetto, S.; Cauda, V.; Civera, P.; Demarchi, D. A Low-Power 0.13-CMOS IC for ZnO-Nanowire Assembly and Nanowire-Based UV Sensor Interface. *IEEE Sens. J.* **2015**, *15*, 4203–4212.
